# Replication of Structured DNA and its implication in epigenetic stability

**DOI:** 10.3389/fgene.2015.00209

**Published:** 2015-06-16

**Authors:** Valentina Cea, Lina Cipolla, Simone Sabbioneda

**Affiliations:** Istituto di Genetica Molecolare, Consiglio Nazionale delle Ricerche, Pavia, Italy

**Keywords:** DNA replication, G4 quadruplex DNA, translesion DNA synthesis, helicases, epigenetic stability

## Abstract

DNA replication is an extremely risky process that cells have to endure in order to correctly duplicate and segregate their genome. This task is particularly sensitive to DNA damage and multiple mechanisms have evolved to protect DNA replication as a block to the replication fork could lead to genomic instability and possibly cell death. The DNA in the genome folds, for the most part, into the canonical B-form but in some instances can form complex secondary structures such as G-quadruplexes (G4). These G rich regions are thermodynamically stable and can constitute an obstacle to DNA and RNA metabolism. The human genome contains more than 350,000 sequences potentially capable to form G-quadruplexes and these structures are involved in a variety of cellular processes such as initiation of DNA replication, telomere maintenance and control of gene expression. Only recently, we started to understand how G4 DNA poses a problem to DNA replication and how its successful bypass requires the coordinated activity of ssDNA binding proteins, helicases and specialized DNA polymerases. Their role in the resolution and replication of structured DNA crucially prevents both genetic and epigenetic instability across the genome.

## Introduction

**FIGURE 1:**
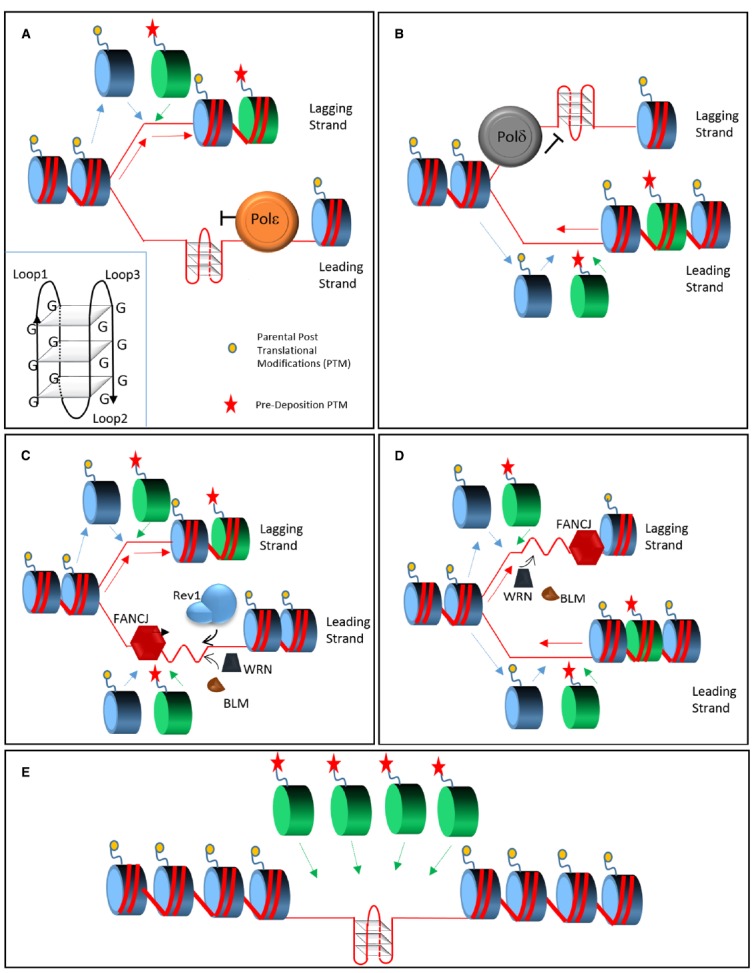
**Model of replication across structured DNA.** During replication, polymerases *ε* and δ stall in presence of a G4 quadruplex (inset), respectively on the leading **(A)** or lagging strand **(B)**. Parental histone recycling (dashed blue lines) continues on the opposing strand supplemented by newly synthetized histone carrying pre-deposition marks (green dashed line). FANCJ coordinates two independent pathway in order to allow G4 bypass. On the leading strand **(C)** FANCJ and Rev1 destabilize the quadruplex from opposing directions; on the lagging strand FANCJ is supported by the action of WRN and BLM **(D)** that may also play a minor role on the leading strand. In presence of the activity of the FANCJ, Rev1, WRN, and BLM **(C,D)** the quadruplex is efficiently replicated without perturbing the recycling and deposition of histones. In absence of these proteins an un-replicated gap, either on the leading or lagging strand, is left behind the fork **(E)**. The gap may be the result of a re-priming event and in this case it will be replicated by a different mechanism, such as Post Replication Repair (not shown). This form of bypass will lead to the loss of epigenetic information since only new histones, without parental post translational modifications, will be available.

DNA replication is a central process in cellular life. Its completion requires both the propagation of the genetic material and the correct transmission of the epigenetic information (Groth et al., 2007; Corpet and Almouzni, 2009). These two activities need to be tightly regulated and coordinated. During fork progression the epigenetic modifications carried by histones are distributed by the histone chaperones Asf1 and Caf1 between parental and daughter strands of the DNA (Gurard-Levin et al., 2014). This mechanism allows local recycling of modified parental histones and incorporation of new histones that are devoid of parental modifications but instead carry pre-deposition marks. This process needs stable and ongoing DNA replication and it could be affected by any event able to perturb DNA duplication. Indeed, acute replication stress affects histone dynamics and alters binding to histone chaperones (Jasencakova et al., 2010). The vertebrate genome is scattered with sequences that can fold in secondary structures and that are for this reason difficult to replicate. Among such sequences, G-quadruplexes are one of the most studied (Maizels and Gray, 2013). A G-rich sequence (G_+3_N_1–7_G_+3_N_1–7_G_+3_N_1–7_G_+3_) has the ability to form a quadruplex (G4) stabilized by Hoogsteen hydrogen bonds at physiological salt concentration (Figure 1A, inset; Sen and Gilbert, 1988). This occurs especially during DNA transitions (replication and transcription) that use ssDNA as an intermediate (Phan and Mergny, 2002; Maizels and Gray, 2013). In such conditions, four guanines can arrange in a quadruplex that is thermodynamically more stable than B-form DNA and it can block DNA replication and transcription both *in vitro* and *in vivo* (Maizels and Gray, 2013). It is estimated that in the genome over 350,000 sequences have the potential to fold in G-quadruplexes that can act as a replication barrier even in unchallenged conditions (Huppert and Balasubramanian, 2005; Todd et al., 2005). While G4 formation was studied *in vitro* for a number of years, their occurrence *in vivo* has only been observed recently. Data collected with G4 recognizing molecules or antibodies (Rodriguez et al., 2012; Biffi et al., 2013; Henderson et al., 2014) show that G4s are present in cells and they increase during S phase. G-quadruplexes have been involved in a variety of biological processes including maintenance of the telomeric regions, control of gene expression, immune response and initiation of DNA replication (Maizels and Gray, 2013). Out of the 250,000 putative replication origins identified in human over 67% are in proximity of a G-quadruplex (Besnard et al., 2012). Recent studies suggest that structured DNA requires replication to form (Biffi et al., 2013) and its presence could hinder DNA duplication. G4 DNA is able to block primer extension *in vitro* (Kamath-Loeb et al., 2001) and it can lead to genomic instability and to the formation of G4 fragile sites in both *S. cerevisiae* and *C. elegans* (Kruisselbrink et al., 2008; Lopes et al., 2011).

Historically a number of helicases have been involved in the resolution of G4 DNA: Pif1 (Sanders, 2010; Schuldt, 2011), the RecQ helicases BLM and WRN (Brosh et al., 2001; Johnson et al., 2010; Indig et al., 2012) and the Fanconi Anemia protein FANCJ (Wu et al., 2008; Bharti et al., 2013). More recently, specialized DNA polymerases have been uncovered to play a new role in replicating past structured DNA (Sarkies et al., 2010).

This review focuses on how G4 DNA is resolved during replication. We will first concentrate on how ssDNA binding proteins can destabilize the formation of G4s and then analyze the helicases that can resolve such secondary structures. We will then investigate the involvement of DNA polymerases that can use G4s as a template during replication and the repercussion of the timing of G4 bypass on maintenance of the genetic and epigenetic information.

## Prevention and Resolution of the G4 Problem

Hoogsteen bond formation is favored in the presence of single stranded DNA. In cells, single strand binding proteins, such as RPA (Replication Protein A) and the telomeric protein POT1, readily bind ssDNA (Wold, 1997; Palm and de Lange, 2008) preventing the formation of structured DNA.

Additionally, these proteins could help to disrupt folded G4s. RPA is the most abundant of the ssDNA binding proteins and it can initially bind a G4 on a three nucleotide loop/overhang via one of its ssDNA binding domains (DBD-A; Qureshi et al., 2012; Ray et al., 2013). The G4 starts to destabilize when the DBD-B interacts with the loop and it is unfolded after the final binding of the DBD-C and DBD-D domains.

The telomeres, ending with a short 50–200 nt long 3′ overhang of ssDNA (Makarov et al., 1997), are regions of tandem repeats prone to form G-quadruplexes. POT1 and TPP1 protect the telomeres and prevent the activation of the DNA damage response mediated by RPA and ATR (d’Adda di Fagagna et al., 2003). The presence of quadruplexes favors the binding of the less abundant POT1/TPP1 complex and protects the overhangs from RPA recognition. Biochemical evidence suggests that POT1, enhanced by TPP1, binds the 3′ end of the telomeric overhang and it unfolds the G4 preventing at the same time RPA access (Ray et al., 2014). The binding of POT1/TPP1 appears to be dynamic and the complex exhibits a movement on ssDNA similar to a sliding clamp (Hwang et al., 2012).

RPA and POT1 may prevent the formation of G-quadruplexes, but once structured regions of DNA are established other proteins need to process them. A group of helicases is involved in the resolution of G4 intermediates: among them two members of the RecQ family, BLM and WRN, the Fanconi Anemia protein J (FANCJ), CHL1 and PIF1. A deficiency in either BLM or WRN is the cause of two genetic diseases called respectively Bloom and Werner Syndrome (Monnat, 2010). They have very different clinical presentations but they are both extremely cancer prone. BLM and WRN work in different DNA repair pathways and they can resolve a number of DNA intermediates like Holiday Junctions or D-loops. Moreover, they can stabilize the replication fork in presence of genotoxic reagents such as Hydroxyurea (Sidorova et al., 2008). WRN can bind its substrates with high affinity while BLM, at least *in vitro*, shows a reduced binding capability. G-quadruplexes are the only exception where BLM binds more efficiently than WRN (Kamath-Loeb et al., 2012). Regardless of these differences, both helicases can resolve all the mentioned structures *in vitro*. BLM and WRN, in addition to the helicase (3′ to 5′) domain, possess two additional distinctive domains called RQC (RecQ C-terminal) and HRDC (Helicase and RNase-D C-terminal; Monnat, 2010). The RQC shows a strong affinity for G4 DNA and the HRDC facilitates substrate recognition (Huber et al., 2006; Chatterjee et al., 2014). In addition, the HRDC domain of BLM is required for binding and resolution of Holiday Junctions (Wu et al., 2005). Initial observations suggested that WRN and BLM could unwind G4s *in vitro* (Sun et al., 1998; Kamath-Loeb et al., 2012). New single molecule studies revealed that this unfolding activity was ATP independent and it relied on specific characteristics of the G4 to be resolved (Budhathoki et al., 2014). In particular BLM activity required a 3′ overhang longer than seven nucleotides and a spacer region, between the G4 and dsDNA, greatly enhanced unfolding of the quadruplex (Chatterjee et al., 2014).

Sequences predicted to form G4s are found often near the transcriptional start site (TSS) of a gene. Interestingly, changes in transcription profiles of such genes have been detected in BLM and WRN cell lines (Johnson et al., 2010; Nguyen et al., 2014), due to transcriptional regulation near the G4.

FANCJ is another helicase involved in G4 resolution. FANCJ is an iron sulfur (Fe-S) DNA helicase involved in Intra-strand Crosslink repair (ICL) as part of the Fanconi Anemia repair pathway (Brosh and Cantor, 2014). Fanconi Anemia (FA), a disease that presents congenital defects, bone marrow failure and predisposition to tumors, affects patients carrying mutations in FANCJ. Differently from other members of the Fe-S helicase family (XPD, RTEL, and DDX11) FANCJ is the only one capable of unwinding G4 DNA *in vitro* (Wu et al., 2008; Bharti et al., 2013). *In vivo*, the quadruplex stabilizing drug telomestatin causes DNA damage in FANCJ deficient cells leading to DSBs and accumulation of γH2AX (Bharti et al., 2013). In *Xenopus Laevis* egg extracts, FANCJ promotes bypass on a plasmid carrying a G4 and it prevents replication stalling (Castillo Bosch et al., 2014) in a manner independent from the other Fanconi proteins. In *C. elegans*, DOG-1, the FANCJ homolog, appears to protect cells from genome instability at G4 DNA sites. Worms mutated in DOG-1 show instability near G4 forming sequences and accumulation of small deletions (Kruisselbrink et al., 2008; Maizels, 2008). These deletions are generated by an end-joining repair pathway mediated by the DNA polymerase theta (Koole et al., 2014; van Kregten and Tijsterman, 2014). Indeed deletion of POLQ exacerbates G4 instability in a *dog-1* background. Overall, we can conclude that helicases and ssDNA binding proteins protect the cells from accumulation of structured DNA that would be detrimental for their metabolism.

## Replicating Across G-quadruplexes via DNA Translesion Synthesis

While the role of ssDNA binding proteins and helicases suggests that unfolding is essential to allow replication of the structured region, recent experimental evidence indicates that specialized DNA polymerases could also be involved in structured DNA bypass. The Y family polymerases polη, polι, polκ, Rev1, and the B family dimer Rev3-Rev7 are already known to be involved in the bypass of distorted templates caused by DNA damage. Together they are part of a DNA damage tolerance pathway called DNA Translesion synthesis (TLS; Sale et al., 2012). TLS polymerases, by virtue of a larger catalytic site, can accommodate damaged templates and they can incorporate nucleotides both in an error free and in an error prone manner. Upon encountering DNA damage the replication fork stalls and the replicative clamp PCNA (Proliferating Cell Nuclear Antigen), ubiquitylated by Rad6-Rad18, promotes a switch between replicative and TLS polymerases. Ubiquitylated PCNA (Ubi-PCNA) shows increased affinity for Ubiquitin binding motifs present on TLS polymerases (Kannouche et al., 2004; Bienko et al., 2005, 2010). The timing of damage bypass is still not understood completely. In different model systems, the bypass has been observed either early at the fork (Edmunds et al., 2008; Jansen et al., 2009) or at a later time following re-priming of the replisome (Lehmann, 1972; Lopes et al., 2006). The temporal choice of TLS appears to have different genetic requirements: post-replicative bypass in mammalian cells and *S. cerevisiae* requires Ubi-PCNA and polη but their role at the replication fork is still unclear. In contrast, in chicken DT40 cells the second type of bypass relies on Rev1 (Edmunds et al., 2008). In MEFs, Rev1 was proposed to have a role in both early and late types of bypass (Jansen et al., 2009).

Rev1 is a dCTP transferase (Lin et al., 1999; Haracska et al., 2002) and in DT40 its catalytic activity is dispensable for damage bypass at the fork that instead requires the protein C-terminus (Edmunds et al., 2008). This domain mediates the interaction with other TLS polymerases and for this reason Rev1 is speculated to coordinate the TLS response (Guo et al., 2003; D’Souza and Walker, 2006; Pustovalova et al., 2012). TLS has been studied mostly in the context of DNA damage bypass but recent evidences suggest a more widespread role of these specialized DNA polymerases.

RNAi silencing of polη and polκ can sensitize human cells to telomestatin and it results in double strand breaks formation near a Guanine rich sequence in the c-MYC promoter (Betous et al., 2009). Knock-down of both polymerases increases G4 instability in *dog-1* deficient strains in *C. elegans* (Youds et al., 2006) but their role is currently under debate since null alleles of *polh-1* and *polk-1* failed to recapitulate such phenotype (Koole et al., 2014). A recent paper suggests that polη can bind a G4 substrate *in vitro* (Eddy et al., 2015) and it is capable to replicate across quadruplexes with higher efficiency and fidelity than the catalytic domain of pol*ε*.

In addition, polη appears to be involved in the replication of common fragile sites (CFS), specific regions of the genome that, in some cell types, are characterized by an increased chance of breakage during replicative stress (Rey et al., 2009; Bergoglio et al., 2013). A subset of CFS replicates late during S phase and it shows a low density of replication origins. The slower replication across CFS could lead to polymerase pausing and accumulation of ssDNA ahead of the fork. This substrate, if not annealed correctly, could form non-canonical DNA intermediates similar to G-quadruplexes.

The structure of polη gives some insights on how the polymerase could bypass G4 DNA and other secondary structures. When it was crystallized in presence of a cyclobutane pyrimidine dimer (CPD), the polymerase was able to interact with the incoming DNA via the back of its little finger, using this domain to open secondary structures on the distorted template (Biertumpfel et al., 2010). In such conformation, polη could form a molecular splint capable of forcing the distorted DNA toward B-form. Thus, it is tempting to speculate that a similar mechanism could allow replication across a G-quadruplex.

Rev1 was shown to bind preferentially G4 DNA *in vitro* and to disrupt its formation (Eddy et al., 2014). Analysis of Rev1 structure revealed the presence of a protein side chain (N-digit) that can displace the incoming template base from its active site. Then the displaced template is stabilized by repeated domains within Rev1 (G-loop), ultimately unfolding the G-quadruplex. Altogether, this suggests that TLS could bypass G4s and structured DNA directly or it could be required for repairing the damage caused by sequences difficult to replicate. Regardless of the molecular mechanism, the involvement of TLS, with its distinct temporal requirements, suggests that G4 bypass could have serious repercussion on the stability of the epigenetic information of the regions neighboring the quadruplex.

## G4 Replication and Epigenetic Instability

Histone deposition needs to be synchronized with replication fork progression. During replication, the MCM helicase complex displaces the parental histones that are promptly recycled by the coordinated action of the histone chaperones Asf1 and Caf1. Parental histones, carrying epigenetic information, are distributed between the two daughter strands in addition to being supplemented by the stock of free naïve histones (Figures 1A,B). As a result, each of the two newly synthetized strands of DNA folds on chromatin carrying only part of the epigenetic modifications on their histone tails. Such modifications are copied by chromatin modifying complexes ensuring maintenance of epigenetic memory (Margueron and Reinberg, 2010). The re-deposition of parental histones is local and it is possible only within the time window between histone eviction and recycling. Ultimately, uncoupling histone deposition and DNA replication could alter epigenetic transmission (Jasencakova et al., 2010).

Bypass of G-quadruplexes during active replication would maintain the original epigenetic status (Figures 1C,D) while delayed gap filling by post replicative repair (PRR) would lead to the preferential incorporation of new histones (Sarkies et al., 2010; Figure 1E). Rev1 in chicken DT40 cells appears to coordinate the first type of bypass and its absence affects the epigenetic status of the chromatin (Sarkies et al., 2010).

In *rev1* cells, we can observe both activation and repression of a large number of genes harboring G4 DNA sequences in their proximity. Exemplary for the first case was the activation of the β-globin locus, normally silent in DT40, with loss of H3K9me2, a modification associated with low gene transcription (Sarkies et al., 2010). Remarkably, an increase in acetylation of H3K9/14 or trimethylation of H3K4 was not observed indicating that the transcriptional state was influenced by the loss of the repressive marker more than the acquisition of an activating one. Conversely, it was possible to appreciate the silencing of CD72 and Bu1a, normally two active genes, with the loss of H3K9/14Ac and H3K4me3, not followed by an increase of H3K9me2 (Sarkies and Sale, 2012). Microarray analysis revealed that 71% of the altered gene expression in *rev1* correlated with the presence of DNA sequences prone to form G-quadruplex structures. Alterations in the histone post-translational modifications could be detected only when the quadruplex was on the leading strand within 4.5 Kb of the TSS, hinting that the epigenetic information around the TSS influenced the expression state of a gene (Schiavone et al., 2014). Mutants of Rev1 lacking its C-terminus could not maintain the chromatin status but the absence of Rev1 catalytic activity had a smaller effect.

The partial dependency on the active site suggests that Rev1 could de-stabilize the G4 by adding a dCTP to the structured template, as observed *in vitro*. On the other hand, the involvement of its C-terminus indicates that Rev1 could coordinate the recruitment of other factors. The other TLS polymerases play a minor role in the DT40 system and only small epigenetic alterations at the β-globin and at the Bu1a loci are observed in single *pol*η and *pol*κ knockouts. Furthermore, even the triple *pol*η/*pol*κ/*rev3* mutant does not show changes comparable to the one observed in *rev1* (Wickramasinghe et al., 2015). Thus, the role of Rev1 appears to be independent from the other TLS polymerases and its C-terminus might interact with additional factors involved in DNA replication such helicases and possibly even replicative polymerases.

An analysis of epigenetic instability excluded the involvement of other repair factors with the exception of FANCJ, WRN, and BLM (Sarkies et al., 2012). WRN and BLM appeared to work together, since loss of transcription of Bu1a could be appreciated only in the absence of both genes. The expression changes observed in the *wrn/blm* mutant were more complex than the ones previously reported in Werner and Bloom cell lines derived from patients. The DT40 system showed an even increase of both up-regulated and down-regulated genes (Sarkies et al., 2012) while the human cell lines predominantly up-regulated transcription (Johnson et al., 2010). Furthermore, the number of transcripts altered in the DT40 double mutant was higher than the single *wrn* and *blm* possibly indicating more than a transcriptional regulation.

Transcription profiling revealed overlaps between the expression changes of the *fancj* mutant with the *rev1* and *wrn/blm* cell lines respectively.

Alterations of gene expression correlated with the presence of G4 DNA near the transcribed loci, pointing to a central role of FANCJ in G4 bypass. FANCJ was also shown to block spreading of irreversible heterochromatisation in the presence of telomestatin (Schwab et al., 2013). In this case, FANCJ was proposed to prevent fork stalling on the lagging strand. Indeed FANCJ deficient cells accumulated stretches of ssDNA of 250–3000 nucleotides, a size compatible with Okazaki fragments. Interestingly, gap filling by PRR results in fragments of 800-1600 nucleotides in mouse cells (Lehmann, 1972) and gaps of similar size (2–3 Kb) are observed on the leading strand behind the fork in yeast (Lopes et al., 2006). Thus, it is difficult to discriminate if FANCJ can prevent fork stalling only on the lagging strand or it could work on both strands. WRN and BLM have been shown previously to help in replicating telomeres formed by lagging strand synthesis (Crabbe et al., 2004; Zimmermann et al., 2014). Nevertheless Sarkies et al. (2012) showed that a plasmid carrying a G4 on the leading strand was not efficiently replicated in a *wrn/blm* double mutant.

It is tempting to speculate that FANCJ could control two bypass pathways mediated respectively by Rev1 and BLM/WRN (Figures 1C,D). FANCJ and BLM/WRN show different helicase directionality and their convergence from opposite sides could unfold the quadruplex (Figures 1C,D). Interestingly BLM and WRN, but not FANCJ, can unfold an intramolecular G4 by simply binding its 3′ overhang in absence of ATP or catalytic activity (Budhathoki et al., 2014; Chatterjee et al., 2014). This suggests that while a 5′ to 3′ activity may be required, WRN and BLM could help FANCJ by interacting with the G4 from the opposite end (Figure 1C).

In the case of Rev1, the polymerase could destabilize the quadruplex by binding it or by inserting a dCTP, before resolution by the incoming FANCJ helicase (Figure 1D). Rev1 activity may be confined to the leading strand while WRN/BLM could act as a backup on the leading but exert a major role on the lagging strand. Overall, both processes would ensure the continuation of DNA replication and the formation of chromatin carrying the parental epigenetic information.

## Concluding Remarks

G4 duplex formation has been detected *in vitro* for a number of years but their presence *in vivo* has always been source of much speculation. Only recent experimental evidence unveiled the existence of G-quadruplexes *in vivo* particularly during the S phase of the cell cycle, with all the consequences that their presence entails. In this short review, we tried to present the latest experimental details showing how G4 structures are bypassed and what proteins are involved in the process. A combination of helicases, including FANCJ, WRN and BLM, is proposed to act on the structured DNA with the help of TLS polymerases. Together these proteins ensure the progression of the replication fork and allow the correct deposition of histones with their epigenetic information. In their absence, part of the epigenetic memory is lost with far reaching consequences at the level of the transcriptional program of the cells.

### Conflict of Interest Statement

The Associate Editor, Alessandra Montecucco, declares that, despite being affiliated with the same institute as the authors Valentina Cea, Lina Cipolla, and Simone Sabbioneda, the review process was handled objectively. The authors declare that the research was conducted in the absence of any commercial or financial relationships that could be construed as a potential conflict of interest.
